# West Nile virus in California, 2003–2018: A persistent threat

**DOI:** 10.1371/journal.pntd.0008841

**Published:** 2020-11-18

**Authors:** Robert E. Snyder, Tina Feiszli, Leslie Foss, Sharon Messenger, Ying Fang, Christopher M. Barker, William K. Reisen, Duc J. Vugia, Kerry A. Padgett, Vicki L. Kramer

**Affiliations:** 1 California Department of Public Health, Vector-Borne Disease Section, Richmond and Sacramento, California, United States of America; 2 California Department of Public Health, Division of Communicable Disease Control, Richmond, California, United States of America; 3 Department of Pathology, Microbiology & Immunology, School of Veterinary Medicine, University of California, Davis, California, United States of America; Louisiana State University, UNITED STATES

## Abstract

The California Arbovirus Surveillance Program was initiated over 50 years ago to track endemic encephalitides and was enhanced in 2000 to include West Nile virus (WNV) infections in humans, mosquitoes, sentinel chickens, dead birds and horses. This comprehensive statewide program is a function of strong partnerships among the California Department of Public Health (CDPH), the University of California, and local vector control and public health agencies. This manuscript summarizes WNV surveillance data in California since WNV was first detected in 2003 in southern California. From 2003 through 2018, 6,909 human cases of WNV disease, inclusive of 326 deaths, were reported to CDPH, as well as 730 asymptomatic WNV infections identified during screening of blood and organ donors. Of these, 4,073 (59.0%) were reported as West Nile neuroinvasive disease. California’s WNV disease burden comprised 15% of all cases that were reported to the U.S. Centers for Disease Control and Prevention during this time, more than any other state. Additionally, 1,299 equine WNV cases were identified, along with detections of WNV in 23,322 dead birds, 31,695 mosquito pools, and 7,340 sentinel chickens. Annual enzootic detection of WNV typically preceded detection in humans and prompted enhanced intervention to reduce the risk of WNV transmission. Peak WNV activity occurred from July through October in the Central Valley and southern California. Less than five percent of WNV activity occurred in other regions of the state or outside of this time. WNV continues to be a major threat to public and wild avian health in California, particularly in southern California and the Central Valley during summer and early fall months. Local and state public health partners must continue statewide human and mosquito surveillance and facilitate effective mosquito control and bite prevention measures.

## Author summary

West Nile virus (WNV) was first detected in the United States in 1999, and subsequently spread throughout the country. It is now the most commonly reported disease that is transmitted by mosquitoes in the United States. California has reported approximately 15% of all human WNV infections since the virus was first reported in the state in 2003. The California Department of Public Health works with partners throughout the state to conduct robust surveillance of WNV infections in mosquitoes, dead birds, and sentinel chickens, using these data to direct vector control activity and reduce the risk of WNV transmission to people. This article summarizes sixteen years of human, mosquito, chicken, equine, and dead wild bird surveillance for WNV in California, the most comprehensive description of WNV surveillance data to date. We found that WNV enzootic and human activity varied geographically and in intensity from year-to-year. Almost 95% of all WNV activity in California occurred in the Central Valley and southern California between April and November.

## Introduction

West Nile virus (WNV) is a mosquito-borne flavivirus (*Flaviviridae*) that was introduced into New York City in 1999, rapidly spread throughout the continental United States, and was first detected in California during the summer of 2003 [1–4]. The virus is maintained and amplified in an enzootic transmission cycle involving mosquitoes in the genus *Culex* and various bird species. Humans may become infected through the bite of an infected female mosquito; in California the primary rural vector is *Culex tarsalis*, whereas *Cx*. *pipiens* and *Cx*. *quinquefasciatus* are the principal urban vectors in the northern and southern parts of the state, respectively. Common bird hosts for WNV in California include American crows and other corvids, as well as house finches and house sparrows. Spillover transmission has led to 50,830 reported human cases of WNV disease, inclusive of 2,330 fatalities, and more than 28,000 equine encephalitis cases across the USA since 1999 [5–8]. Herein, we have summarized WNV surveillance data collected and reported in California from 2003 through 2018.

For more than 50 years, California has maintained a mosquito-borne arbovirus surveillance program that began in response to epidemics of St. Louis encephalitis virus (SLEV) and western equine encephalomyelitis virus (WEEV) in the state’s Central Valley [9–11]. Investigation of the WEEV outbreak of 1952, centered in the San Joaquin Valley, showed that climate variation and viral amplification within enzootic cycles could be monitored to provide an early warning for human disease. Shortly thereafter, along with human and equine encephalitis case surveillance, the California mosquito-borne virus surveillance program began to monitor mosquito abundance and test mosquitoes for virus infection statewide as an early warning system for arboviral activity [12]. In 1979, sentinel chickens were added as a means for monitoring avian infections [13]. The current program is a cooperative effort among the California Department of Public Health (CDPH), the University of California, Davis Arbovirus Research and Training Laboratory (DART) [formerly the Center for Vectorborne Diseases, CVEC], as well as local vector control agencies (hereafter VCAs) and public health agencies throughout California [14]. In 2000, anticipating the eventual spread of WNV to California, the program was expanded to include testing for WNV in humans, horses, mosquitoes, and sentinel chickens, as well as monitoring WNV infections in dead wild birds [1]. Compared to many other states, California is fortunate in that 90% of its population lives within the jurisdictional boundaries of the more than 70 VCAs that conduct arboviral surveillance and respond to elevated disease risk with enhanced mosquito control.

In July 2003, WNV was detected for the first time in California in *Cx*. *tarsalis* mosquitoes collected in Imperial County in southern California. Shortly thereafter, WNV-positive mosquitoes and sentinel chickens were reported in Imperial and Riverside counties, and by October, WNV had been detected in more than 50 dead birds representing eight species collected throughout southern California, including the densely populated urban corridor between Los Angeles and San Diego. In late September the first human case of WNV disease was identified in a resident of Riverside County, and ultimately three cases of WNV disease were reported from southern California by the year’s end. In 2004, the virus spread throughout the entire state, with 779 human cases in 23 counties, 540 equine infections in 31 counties, and enzootic transmission in all 58 counties. From 2003 through 2018, WNV activity was detected annually by all surveillance indicators, and more WNV disease was reported to the U.S. Centers for Disease Control and Prevention (CDC) by California than by any other state.

Sequencing and phylogenetic analyses of WNV isolates have shown that the 1999 New York viral isolate from a dead Chilean flamingo (NY99, AF196835) was related closely to a 1998 isolate from a dead goose in Israel (IS-98 STD, AF481864) [15–17]. This Lineage Ia strain contained the NS3-T249P mutation that causes elevated viremia and mortality in American crows [18]. As the virus moved west across the United States, a positively selected envelope gene mutation was acquired in 2001 (NA/WN02) that enhanced transmission by some *Culex* species and this genotype ultimately displaced the initially imported NY99 strain [19,20]. By 2003, a third genotype (SW03) was identified from mosquito isolates collected in Arizona, Colorado, and northern Mexico [21,22]. Both WN02 and SW03 strains have invaded California on multiple occasions since 2003, and currently co-circulate in the state [22].

Many of the previously published analyses of WNV disease in California have been limited in geographic scope [23,24], focused on relatively short time frames [25], or described case-series of WNV disease in humans [26–28]. Most other analyses of WNV in California have been similarly limited, only evaluating surveillance parameters in isolation, or focusing on individual counties, smaller regions, and shorter timeframes [29–39]. We sought to comprehensively summarize statewide WNV surveillance data in California from 2003 through 2018.

## Methods

### Ethics statement

Human subjects’ data were collected routinely by CDPH for public health surveillance. Title 17, Section 2500 of the California Code of Regulations specifies which data must accompany WNV case reports. Human data collected for these purposes are stored in a secure location where access is restricted to appropriate CDPH staff. Analysis of human surveillance data is considered routine public health activity and therefore exempt from Institutional Review Board review and approval.

Enzootic surveillance data are the property of the agencies that generate them. These data were obtained through CalSurv data request #000025 submitted 4/16/2018 to the California Vectorborne Disease Surveillance System [40]. CDPH maintains a permit agreement with California Department of Fish and Wildlife to collect dead bird carcasses for WNV testing in California.

### Human surveillance

Title 17, Section 2500 of the California Code of Regulations mandates reporting of WNV-positive diagnostic test results to the local health department where the patient resides. Health departments then conduct investigations and report cases to CDPH that fulfill the Council of State and Territorial Epidemiologists’ (CSTE) case definition for WNV, which has changed several times since the virus’s introduction to the USA [41]. Prior to 2010, all identified WNV infections were reported to CDPH via a standardized paper form. In 2010, California launched an electronic case management system, the California Reportable Disease Information Exchange (CalREDIE), and by 2018, all but two jurisdictions in the state used CalREDIE to manage and report WNV disease.

From 2003 through 2018, specimens from suspect cases were tested by local public health laboratories, commercial laboratories, and the CDPH Viral and Rickettsial Disease Laboratory (CDPH-VRDL). Specimens (blood components and/or cerebrospinal fluid (CSF)) were tested for WNV with an IgM immunofluorescent assay (IFA) or an IgM enzyme immunoassay (EIA). A handful of symptomatic infections also were identified via a nucleic acid-amplification test (NAT). Confirmatory testing with plaque-reduction neutralization tests (PRNT) was conducted by CDPH-VRDL as necessary. These comparative endpoint PRNTs were recommended when two endemic flaviviruses were circulating simultaneously within a county (i.e., SLEV and WNV), for case-patients with ambiguous EIA or IFA test results, and, when possible, to confirm the first identified case and/or fatality of the year in each county. Diagnostic testing for WNV disease is described more thoroughly elsewhere [42–44].

Asymptomatic WNV infections do not fulfill the CSTE case definition for WNV and therefore were not considered cases in the present analysis [41]. These infections were identified via surveillance performed by blood banks, organ procurement organizations, and the commercial laboratories that serve these entities. Donor samples were tested for viral RNA via NAT, and positive results were reported subsequently to the local health department for investigation. Two weeks post-donation, presumptive viremic blood donors (PVD) were contacted to determine whether symptoms had developed, although this was not always possible. PVD were reclassified as WNV cases per their clinical presentation (i.e. neuroinvasive or non-neuroinvasive) if WNV disease symptoms were reported to investigators after their positive NAT. A small number of additional asymptomatic WNV infections were identified and reported to CDPH during screening for other miscellaneous conditions.

### Mosquito surveillance

Adult mosquitoes were collected by local VCAs using a variety of traps and sampling methods, sorted by species into pools of ≤50 females, and then frozen at -80° C prior to arbovirus testing [45]. Centralized arbovirus testing was conducted by DART, and in recent years this has been augmented with testing by some VCAs. In 2003, all mosquito pools were screened at DART for WNV, WEEV, SLEV, and California group *Bunyaviridae* via in-situ EIAs [45]. From 2004 onward, DART tested mosquito pools for WNV, SLEV, and WEEV via a multiplex real-time (TaqMan) reverse transcriptase-polymerase chain reaction (RT-qPCR) [46]. Pools with cycle threshold scores above 35 which used a primer-probe set targeting the envelope gene, [primers: F, 5'- TCA GCG ATC TCT CCA CCA AAG -3’ & R, 5'- GGG TCA GCA CGT TTG TCA TTG -3’; probe: FAM-TGC CCG ACC ATG GGA GAA GCTC -BHQ1] [47] were confirmed via singleplex RT-qPCR using a second primer-probe set specific to the NS1 region of the genome [primers: F, 5'-GGC AGT TCT GGG TGA AGT CAA -3' & R, 5'-CTC CGA TTG TGA TTG CTT CGT -3'; probe: FAM-TGT ACG TGG CCT GAG ACG CAT ACC TTG T-BHQ1] [48]. In 2005, some VCAs began testing mosquito pools in-house for WNV via RT-qPCR or a rapid commercial antigen-capture assay (RAMP, [Rapid Analyte Measurement Platform, Response Biomedical Co., Vancouver, BC, Canada]). By 2018, twelve VCAs were conducting in-house testing via single or multiplex RT-qPCR, and RAMP testing had been universally discontinued due to its lower sensitivity relative to RT-qPCR [49].

Since 2005, DART has collaborated with CDPH and local VCAs to administer proficiency panels that included both known and blinded diagnostic samples. These results were reviewed and approved each spring by CDPH prior to accepting arboviral testing results from VCA laboratories for subsequent state and national reporting. Commercial rapid assay kits also were evaluated with the proficiency panels to compare sensitivity and specificity to RT-qPCR results. Beginning in 2016, these panels also included blinded tests of simulated mosquito pools in which known WNV inocula of variable titers were spiked into mosquito homogenates. All mosquito collection and virus testing results were reported to CDPH by fax (2003–2005) or electronically via the CalSurv Gateway (2006–2018).

### Dead wild bird surveillance

Since 2000, CDPH has maintained a WNV call center and online reporting system (http://westnile.ca.gov/report_wnv.php) for the public to report dead wild birds [50]. From 2003 through 2018, the call center was operated from mid-April through mid-October. Beginning in 2006, a web-based report form was made available year-round [51]. Participation, criteria, and types of bird species collected varied by VCA and year.

All reports made to the WNV call center or online reporting system were screened by CDPH staff to evaluate if carcasses were compatible with each VCA’s collection criteria. Carcasses fulfilling collection criteria and in acceptable condition (e.g., dead <24 hours, minimal visible decomposition, eligible species, etc.) were collected by VCAs for testing. In 2005, the number and distribution of reported dead birds were used to identify areas with elevated WNV transmission risk, independent of testing intensity [36].

Bird carcasses were tested for WNV at DART or a VCA. From 2003 through 2013, carcasses to be tested by DART were shipped on ice to the California Animal Health and Food Safety (CAHFS) laboratory at the University of California, Davis. These carcasses were necropsied at CAHFS and multiple tissue samples were sent to DART for singleplex RT-qPCR and/or virus isolation using Vero cell culture. Beginning in 2004, oral swabs collected from American Crows and kidney samples from other species were tested via RT-qPCR, using methods identical to mosquito testing [47,52,53]. Carcass necropsies were discontinued in early 2013, and thereafter VCAs collected oral samples via cotton swabs, which then were transferred to nucleic acid preservation cards [54,55]. Preservation cards were shipped to DART, where they were tested by RT-qPCR. From 2004 through 2018, up to 12 VCAs also conducted their own testing of tissue samples and/or oral swabs collected from bird carcasses. Local agencies also tested some birds using a commercial antigen-capture RAMP or VecTOR/VecTest (Medical Analysis Systems, Inc., Camarillo, CA) [54], but this was universally discontinued by 2018.

### Equine surveillance

Title 9 of the California Food and Agriculture Code requires veterinarians and laboratories to report equine cases of WNV to the California Department of Food and Agriculture (CDFA). Serum or brain tissue specimens from horses were tested for WNV at the CAHFS laboratory via an IgM capture ELISA, or by commercial laboratories as described elsewhere [56]. After investigation by CDFA, confirmed infections were reported to CDPH.

### Sentinel chicken surveillance

Participating VCAs obtained hens (~18 weeks old) each spring from commercial producers. Each chicken was banded with a unique number and a baseline blood sample was collected to ensure they were immunologically naive for WNV, WEEV and SLEV. Flocks comprised of 6–10 chickens per coop were usually deployed between April and November at locations chosen by each VCA—typically those with a documented history of arbovirus activity or elevated mosquito abundance. In some years, flocks (≤10 statewide) were retained by select VCAs throughout winter months. Chickens were bled every other week by comb prick, and blood was collected onto individual filter paper strips and tested as described previously [57]. When required for confirmatory testing, or for differentiating WNV from SLEV, whole blood was collected by jugular or brachial venipuncture, centrifuged, and serum was stored at -4° C until tested. Some agencies replaced positive hens to maintain flock size.

Samples were tested for IgY antibodies to WNV, SLEV, and WEEV by EIA at CDPH-VRDL (2003 through 2007) and the CDPH Vector Borne Disease Section (VBDS) laboratory (2008 through 2018) [58]. EIA-positive samples were confirmed by either IFA, western blot, or PRNT [59,60]. PRNT was conducted by VRDL. Up to four VCAs also tested sentinel chicken samples in their local laboratories by EIA; positive samples were sent to VRDL or VBDS for confirmation.

### Non-equine mammal surveillance

Beginning in 2004, dead tree squirrels were reported to the WNV dead bird call center, shipped to CAHFS, and necropsied. Kidney tissue was tested at DART for WNV via RT-qPCR [61]. After 2013, a few agencies paid for non-equine mammal carcasses to be tested at CAHFS, although routine surveillance of these mammals was halted. Other animals have been periodically tested by CAHFS and reported to CDPH, including those housed in zoos or private collections.

### Analyses

SAS 14.3 (SAS Institute, Cary, NC, USA) was used for descriptive analyses, figures, and tables [62]. Maps were made in ArcGIS Pro 2.2.0 (ESRI, Redlands, CA, USA). The number of WNV human infections was summarized by county and clinical presentation (West Nile neuroinvasive disease (WNND), West Nile non-neuroinvasive disease, or asymptomatic WNV infection) for each week, month, and year, in accordance with the Council for State and Territorial Epidemiologists’ case definition for arboviral disease [41]. Asymptomatic infections were defined as infections that were reported to CDPH without symptoms. Due to an inability to verify specific symptoms at the time of analysis, West Nile non-neuroinvasive disease cases with purportedly neuroinvasive symptoms were still considered non-neuroinvasive. Differences in the frequency of clinical complications between neuroinvasive and non-neuroinvasive disease among WNV patients were explored with a chi-squared test.

Subanalyses were performed to describe WNV prevalence by sex, age, race, ethnicity, county of residence, region, and donor (organ or blood) status. Annual disease incidence was estimated per 100,000 persons using population estimates from the California Department of Finance for respective years, as appropriate. Population estimates from 2018 were used to calculate 16-year cumulative incidence [63]. Three regions were grouped spatially based on county geography: 1) the Central Valley: Butte, Colusa, Fresno, Glenn, Kern, Kings, Madera, Merced, Placer, Sacramento, San Joaquin, Shasta, Stanislaus, Sutter, Tehama, Tulare, Yolo, and Yuba counties, 2) southern California: Imperial, Los Angeles, Orange, Riverside, San Bernardino, San Diego, and Ventura counties, and 3) other California: the remaining thirty-three counties.

Disease onset dates (or specimen collection dates among asymptomatic infections and symptomatic infections with missing date of onset) were used to evaluate temporal patterns of WNV disease in California, whereas the dates of mosquito collections, sentinel chicken bleeds, or bird carcass collections were used for temporal analyses of enzootic data.

The minimum infection rate (MIR) for WNV within mosquito pools was calculated as:

$$\frac{positive\;pools\;(N)}{mosquitoes\;tested\;(N)}*1000$$

Among sentinel chickens, flock seroconversion rate was defined as the percentage of flocks with at least one positive chicken.

In 2006, DART adapted a previously existing database into a centralized web-based application to improve laboratory reporting and WNV program operations. Today, the California Vectorborne Disease Surveillance (CalSurv) Gateway is a repository for all mosquito-borne virus surveillance data in California [40], and provides web-based tools for real-time data management, reporting, visualization, and analysis [64]. CalSurv data are stored in a back-end PostgreSQL database with PostGIS for advanced data retrieval and aggregation [60].

## Results

### Human surveillance

**Fig 1:**
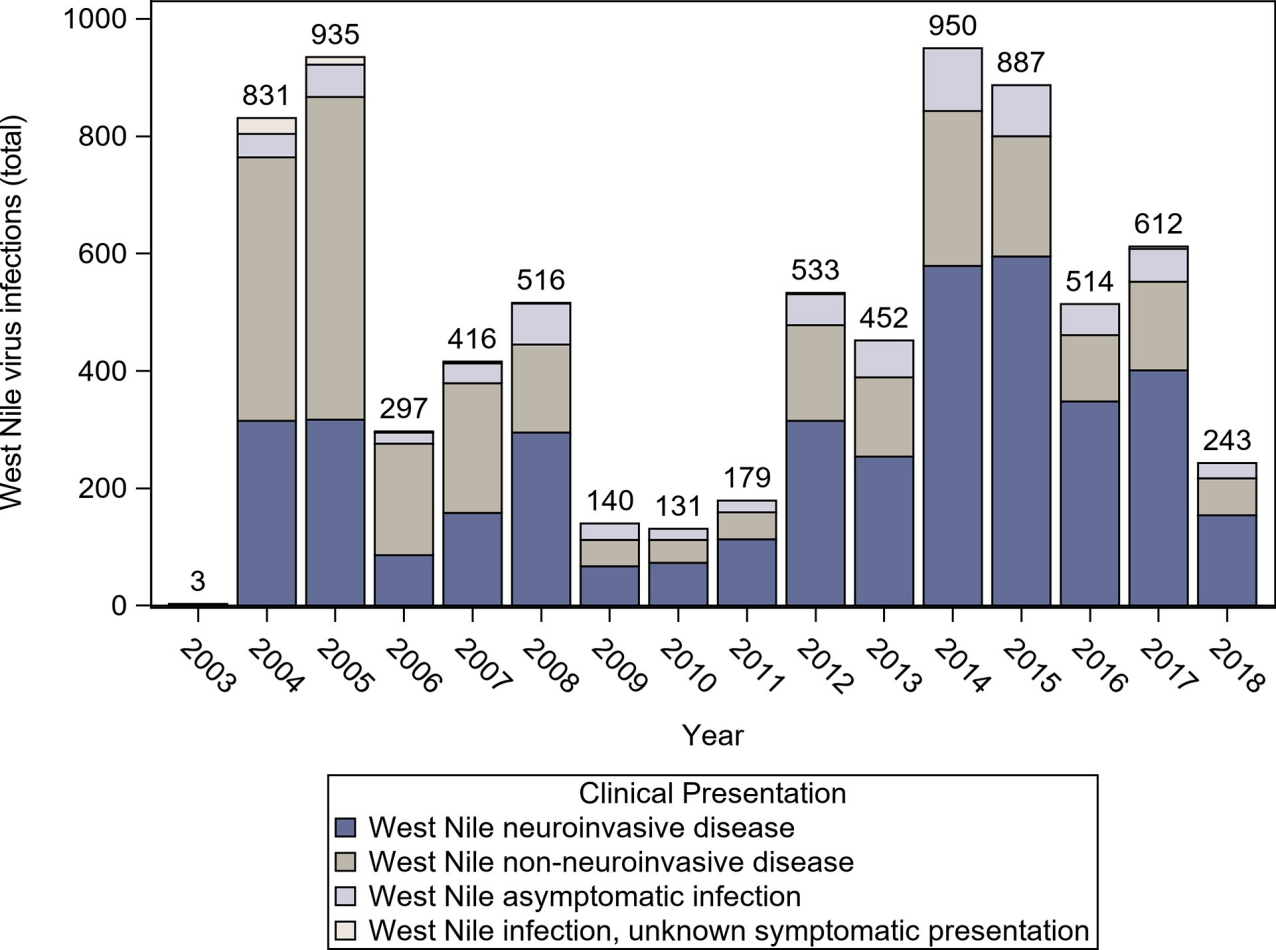
Human West Nile virus infections reported to California Department of Public Health, by clinical presentation, by year, California, 2003–2018.

From 2003 through 2018, 6,909 human cases of WNV disease were reported in California, including 326 WNV-associated fatalities (case-fatality rate = 4.7%). Of these 6,909 cases, 4,073 (59.0%) were WNND, 2,784 (40.3%) were West Nile non-neuroinvasive disease, and 52 (0.8%) had unknown clinical presentations. The number of WNV cases varied by year during this period, with non-neuroinvasive cases being reported more frequently than WNND cases from 2004 through 2007 and WNND cases reported more frequently than non-neuroinvasive cases thereafter (Fig 1). During this time, California reported 15% of all WNV cases in the United States. In addition to these cases, 730 asymptomatic infections also were reported to CDPH, of which 691 (94.5%) were identified through blood donor screening, 8 (1.1%) through organ donor screening, and 31 (4.2%) through other screening as indicated by that patient’s medical provider. There were 117 blood donors (16% of asymptomatic infections) that did not have case investigations conducted who were assumed not to have developed symptoms.

The demographic characteristics of West Nile virus disease case-patients, as well as those of asymptomatic patients are listed in Table 1. More males were reported to be infected with WNV than females. The median age among WNND case-patients was 59 years, 52 among non-neuroinvasive case-patients, and 51 among individuals with asymptomatic infections; 49.2% (2,005 cases) of WNND occurred in patients ≥60 years old. Race information was missing for 34.0% (2,346) of cases and ethnicity information missing for 30.8% (2,125). Among cases whose race or ethnicity was reported, 61.6% (4,256) were reported as white, and 22.7% (1,568) were reported as Hispanic.

**Table 1:** Demographic characteristics of human West Nile virus (WNV) disease cases (n = 6,909) and asymptomatic infections (n = 730) reported to the California Department of Public Health, 2003–2018.

	Cases / Symptomatic WNV infections* (n, %)		Asymptomatic WNV infections (n, %)°
	Neuroinvasive disease	Non-neuroinvasive disease	
**Reported (N)**	4073 (53.3%)	2784 (36.4%)	730 (9.6%)
**Male gender**	2647 (65%)	1538 (55.2%)	408 (55.8%)
**Age in years (median, IQR)**	59 (47, 71)	52 (41, 63)	51 (32, 60)
**0–19**	123 (3%)	128 (4.6%)	52 (8.5%)
**20–39**	521 (12.8%)	519 (18.6%)	142 (23.2%)
**40–59**	1424 (35%)	1234 (44.3%)	265 (43.3%)
≥**60**	2005 (49.2%)	903 (32.4%)	153 (25.0%)
**Race**			
**White**	2550 (62.6%)	1706 (61.2%)	286 (49.4%)
**Other/unknown/not reported**	1339 (32.9%)	1007 (36.2%)	307 (50.0%)
**Asian/Pacific Islander**	102 (2.5%)	46 (1.7%)	13 (2.5%)
**African American**	82 (2.0%)	25 (1.0%)	8 (1.5%)
**Ethnicity**			
**Hispanic**	1131 (27.8%)	437 (15.7%)	83 (13.5%)
**Non-Hispanic**	1762 (43.3%)	1239 (44.5%)	238 (38.7%)
**Unknown**	1017 (25.0%)	1108 (39.8%)	293 (47.7%)
**Region**			
**Southern California**	2673 (65.6%)	1174 (42.2%)	403 (55.3%)
**Central Valley**	1253 (30.7%)	1528 (54.9%)	291 (39.8%)
**Other California**	147 (3.6%)	82 (2.9%)	36 (4.9%)
**Donor Status**			
**Blood donor screening°**	24 (0.6%)	244 (8.8%)	691 (94.5%)
**Organ donor screening**	2 (<0.1%)	1 (<0.1%)	8 (1.1%)

IQR–interquartile range.

* There were 52 infections (0.7% of all infections) reported to CDPH that were symptomatic, but the clinical manifestation was not determined due to the absence of patient interview and/or medical records in 2004 (27), 2005 (13), 2006 (2), 2007 (3), 2008 (1), 2012 (2), and 2017 (4).

° There were 117 blood donors reported that did not have complete case investigations. These reports did not include demographic data, and were excluded from summaries of gender, age, race, and ethnicity. Importantly, these individuals were not contacted to query whether symptoms developed.

**Fig 2:**
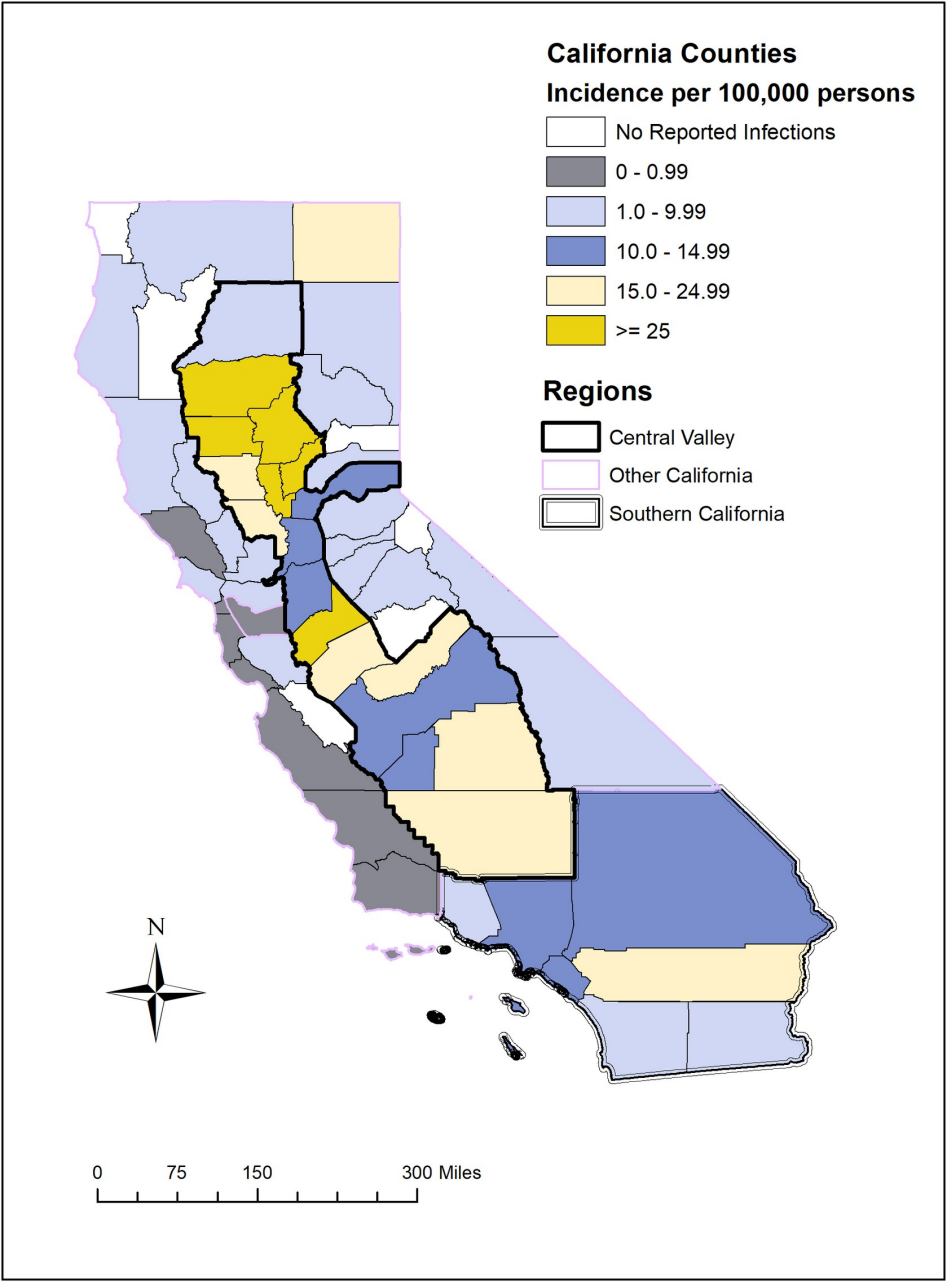
West Nile neuroinvasive disease cumulative incidence per 100,000 persons, by county, California, 2003–2018.

Although WNV disease varied in its geographic distribution from year-to-year, the 16-year cumulative incidence was highest in the Central Valley, followed by southern California, and other California (45.2, 19.1, and 4.0 infections / 100,000 persons, respectively) (Fig 2). Sparsely populated Glenn County, situated within the rice growing region of the northern Central Valley, had the highest cumulative incidence of WNND and total disease incidence (neuroinvasive and non-neuroinvasive) among all California counties with 97.2 and 305.6 cases / 100,000 persons, respectively. However, during most years, densely populated southern California had the most reported cases. Six counties did not report any human WNV infections: Alpine, Del Norte, Mariposa, San Benito, Sierra, and Trinity (S1 Table).

The vast majority, 95.3% (3,881), of WNND case-patients were hospitalized, compared with only 36.8% (1,026) of non-neuroinvasive case-patients (p < 0.05) (Table 2). Approximately 83% (3,366) of WNND case-patients reported a febrile illness, compared with 75.2% (2,093) of non-neuroinvasive case-patients (p < 0.05). The majority of both WNND and non-neuroinvasive case-patients reported headaches (2,480, 60.9%; 1,840, 66.1%, respectively), whereas 54.1% (2,202), 48.9% (1,992), and 34.9% (1,423) of WNND case-patients reported meningitis, altered consciousness, or encephalitis, respectively (p < 0.05 for all except headache, when comparing the frequency of these clinical presentations between neuroinvasive and non-neuroinvasive case-patients). Ten case-patients were reported as non-neuroinvasive, despite having developed either meningitis (6, 0.2%) or encephalitis (4, 0.1%), whereas 328 (11.8%) case-patients with West Nile non-neuroinvasive disease reported altered consciousness. Rash was reported in 37.6% (1,049) of West Nile non-neuroinvasive disease case-patients compared to only 15.7% (641) of those with WNND (p < 0.05).

**Table 2:** Clinical characteristics of human West Nile neuroinvasive disease (n = 4,073) and non-neuroinvasive disease cases (n = 2,784) reported to the California Department of Public Health, 2003–2018.

	West Nile neuroinvasive disease (n, %)	West Nile non-neuroinvasive disease (n, %)
**Hospitalized**°		
**Yes**	3881 (95.3%)	1026 (36.8%)
**No**	126 (3.1%)	1385 (49.8%)
**Unknown**	66 (1.6%)	373 (13.4%)
**Clinical presentation**		
**Febrile illness**°	3366 (82.6%)	2093 (75.2%)
**Headache**	2480 (60.9%)	1840 (66.1%)
**Meningitis**°	2202 (54.1%)	6 (0.2%)
**Altered consciousness**°	1992 (48.9%)	328 (11.8%)
**Encephalitis**°	1423 (34.9%)	4 (0.1%)
**Stiff neck**°	1375 (33.8%)	774 (27.8%)
**Rash**°	641 (15.7%)	1049 (37.6%)
**Seizure**°	208 (5.1%)	16 (0.6%)
**Pregnant**	14 (0.3%)	2 (0.2%)
Death attributed to WNV*°	303 (7.4%)	20 (0.7%)

* Three fatalities occurred in cases that did not report clinical presentation.

° Statistically significantly different frequency of presentation of clinical complication (p < 0.05).

**Fig 3:**
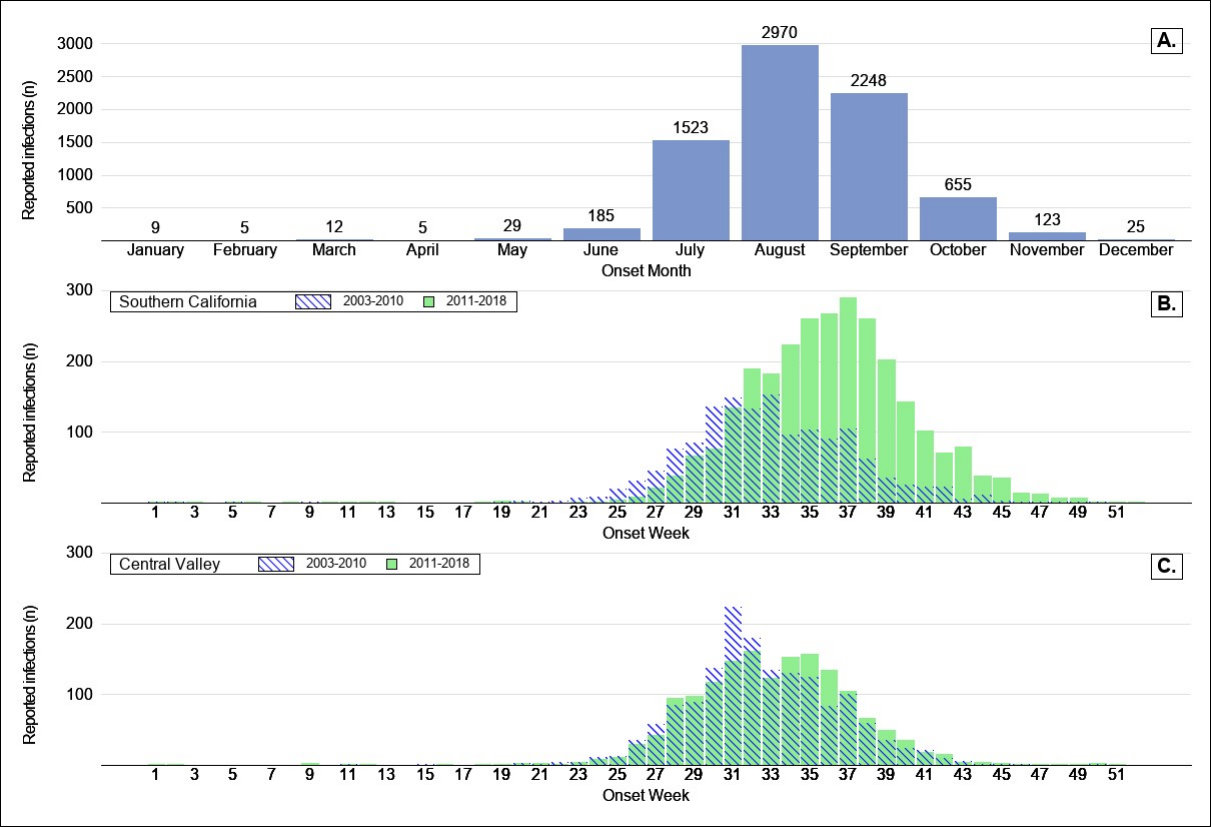
Reported human West Nile virus infections in California, 2003–2018, A) by month of onset date, and by onset week, stratified by year of occurrence in B) Southern California and C) California’s Central Valley*.

Most WNV cases (92.6%, 6,347) developed symptoms between July and October, with an additional 164 and 97 reported in June and November, respectively (3.8% combined) (Fig 3). More cases reported symptom onset in August (2,888, 38.2%) than in any other month, followed by September (2,191, 29.0%), and July (1,467, 19.4%). Only 249 cases reported symptom onset between December 1 and May 31 (3.6%).

WNV disease cases in southern California during 2003–2010 peaked earlier in the year than cases during 2011–2018 (Fig 3). In contrast, the peak of reported cases in the Central Valley remained relatively consistent during both of these time periods. This trend did not appear to be driven by aberrant outbreak years in which clusters of cases occurred earlier (or later) during one or two years compared with the other years.

### Mosquito surveillance

**Fig 4:**
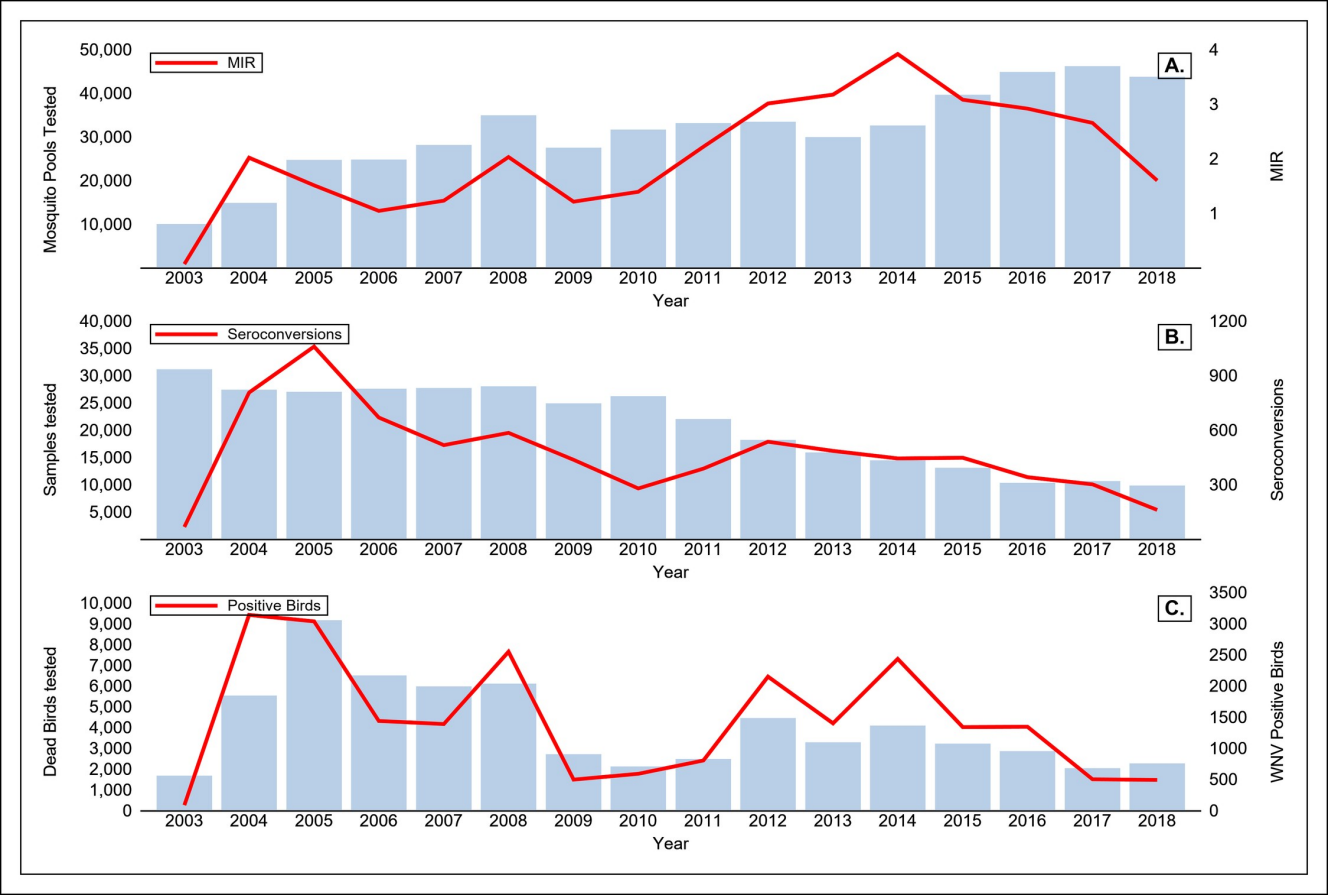
West Nile virus (WNV) enzootic activity in California, 2003–2018 in A) mosquito pools tested with WNV minimum infection rates (MIR) per 1,000 tested B) sentinel chickens tested with number of seroconversions, and C) dead wild birds tested with the number that were WNV positive.

From 2003 through 2018, adult mosquitoes were submitted for WNV testing from an average of 37 counties each year. Among 503,636 mosquito pools that were comprised of 14,515,703 total mosquitoes, WNV was detected in 31,695 (6.3%) pools from 43 (74%) of 58 counties (S2 and S3 Tables). The annual number of pools tested increased more than four-fold from 2003 through 2018 (Fig 4), with 96% tested by RT-qPCR and, in earlier years, 4% by RAMP. Forty-two mosquito species from seven genera were tested for WNV (S4 Table). WNV was detected in 17 species within four genera, but 97.8% of all positive pools were comprised of *Cx*. *pipiens*, *Cx*. *quinquefasciatus*, or *Cx*. *tarsalis*.

**Fig 5:**
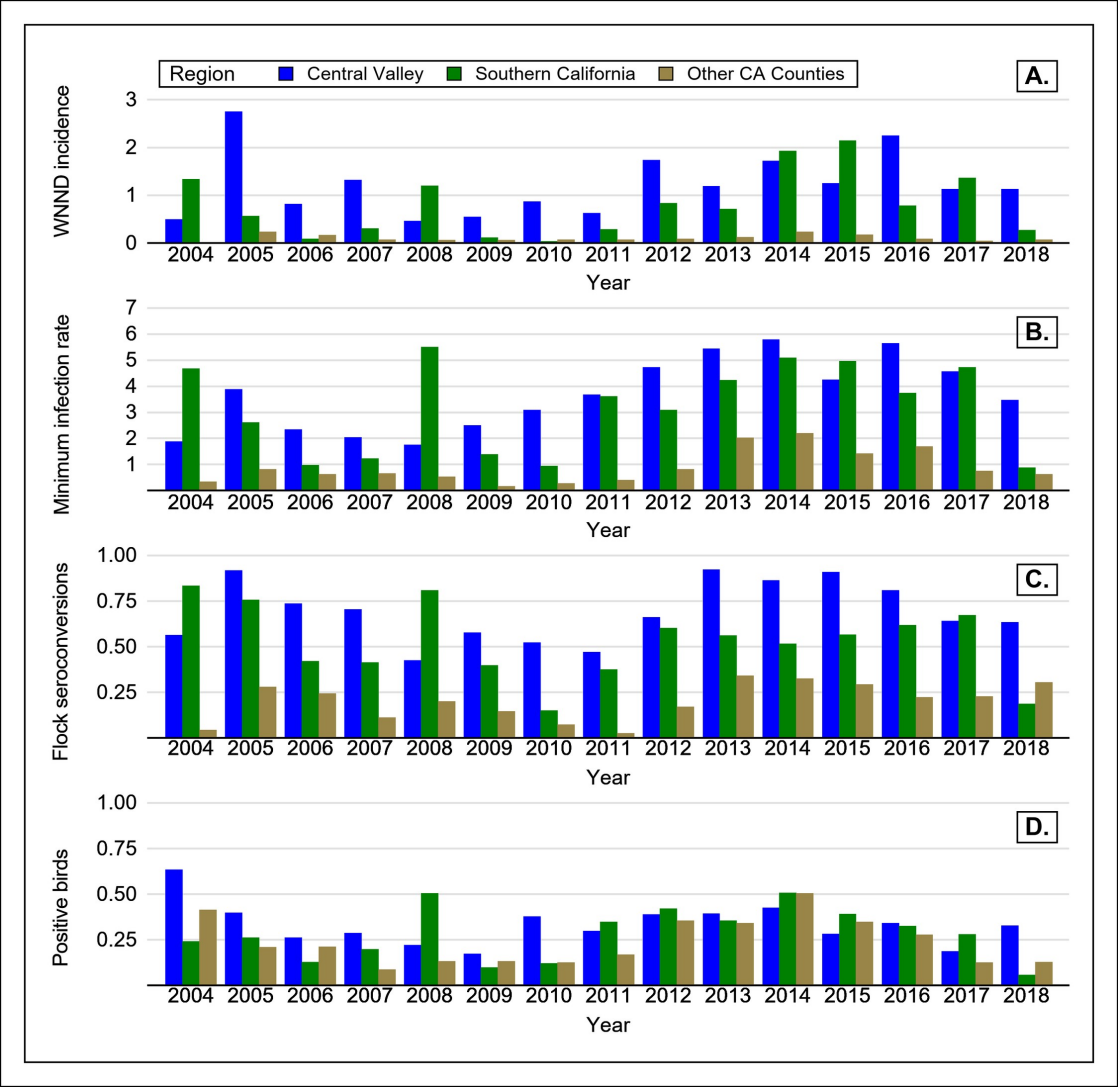
West Nile virus activity by year and region in California, 2004–2018, A) West Nile neuroinvasive disease incidence per 100,000 persons (annual), B) mosquito minimum infection rate per 1,000 mosquitoes tested (July-October), C) proportion of sentinel chicken flocks that seroconverted (July-October) and D) proportion of tested dead wild birds positive for WNV (July-October).

Excluding 2003, the annual statewide WNV MIR among *Culex spp*. ranged from 1.11 (2006) to 3.99 (2014). S3 Table shows the MIR among *Culex spp*. by county and year in California. Fig 4 shows the WNV MIR among all mosquito species. After the initial detection of WNV in 2003, WNV-positive mosquito pools were detected annually in all three regions, although the MIR varied by region and year (Fig 5). The first positive mosquitoes were detected in southern California, except in 2009 and 2013. WNV-positive mosquito pools were detected as early as January 10 (2007, Riverside County) and as late as December 14 (2016, Orange County). The average earliest WNV-positive mosquito detections were on March 24, May 22, and June 8 in southern California, the Central Valley, and other California, respectively.

Ninety percent of all positive pools were collected between July and October, with most collected in the first week of August (3,054, 9.6%). The annual MIR from July 1 through October 31 was highest in the Central Valley region every year except 2004, 2008, 2015, and 2017, and ranged from 1.76 (2008) to 5.8 (2014) per 1,000 female mosquitoes tested (Fig 5). WNV was not detected in mosquitoes collected from Monterey or San Francisco counties.

### Dead bird surveillance

From 2003 to 2018, 450,455 dead birds were reported to the CDPH call center (67%), online (26%), or directly to local VCAs (7%). Of these, 64,829 (14.4% of reports) were tested for WNV, and 23,322 (36.0% of birds tested) were positive from all 58 counties in California (Fig 4, S5 Table). One hundred and eighty-three different bird species were found dead and tested positive for WNV, including passerines, raptors, seabirds, waterfowl, doves, woodpeckers, hummingbirds, and exotic parrots (S6 Table) [53 and C. Barker, personal communication]. The vast majority (81%) of WNV-positive birds were corvids. Among WNV-positive birds, 56% (13,154) were American crows (*Corvus brachyrhynchos*), 17.4% (4,048) were California scrub jays (*Aphelocoma californica*) and 5.7% (1,337) were yellow-billed magpies (*Pica nuttalli*) (S6 Table). Among WNV-positive dead corvids, the species with the highest percentage positive was the yellow-billed magpie (1,337 positive/1,883 tested, 71%), whereas the oak titmouse (*Baeolophus inornatus*) had the highest percentage of tested birds that were positive (31/67, 46.2%) among non-corvid species. Red-tailed hawks (*Buteo jamaicensis*) had the highest percentage positive for WNV among raptors (99/413, 24%).

From 2004 to 2018, the annual percentage of WNV infections detected in tested dead birds ranged from a low of 18.5% (2009) to a high of 59.4% (2014). On average, the first WNV-positive dead bird was reported on January 16. Annually, the first WNV-positive dead bird occurred in southern California from 2004 through 2011 as well as in 2016 and 2017. In 2012, 2013, and 2014 the first WNV-positive dead birds were detected in the Central Valley, whereas in 2015 and 2018, the first detections were elsewhere in California. WNV-positive dead birds have been detected every month of the year, although most (19,076, 82.1%) were found from July through October, with only 6.8% in October. More positive dead birds were reported in August (5,700, 31.1%) than any other month. Although only a few dead birds were reported or collected during the winter months of December, January, February, and March (10.2% of total), 256 (1%) birds tested positive for WNV in these winter months, suggesting the possibility of continued viral transmission during the winter. Southern California had the highest percentage of positive dead birds among those tested (42%), followed closely by the Central Valley (35%) (Fig 5).

### Equine surveillance

In 2003, a single equine case was identified in a 20-year-old unvaccinated horse in San Diego County. In 2004 and 2005, 996 cases were reported from 43 counties, leading to 429 fatalities (CFR = 43.1%). However, from 2006 through 2018, only 303 equine cases and 105 fatalities (CFR = 34.5%) were reported to CDPH. From 2003 through 2018, Riverside County reported the most equine infections (170, 13.2% of equine cases), followed by Sacramento County (136, 10.5%). The majority, 64.2%, of equine infections were in the Central Valley, 23.8% were in southern California, and 11.9% were in other California. Four counties (6.9%) have never reported any equine WNV infections: Del Norte, Inyo, San Francisco, and San Mateo counties.

### Sentinel chicken surveillance

From 2003 through 2018, sentinel chicken flocks were maintained in an average of 35 counties each year (60% of CA counties; range, 25–40). The number of flocks statewide ranged from 110 (2018) to 262 (2005) (S7 Table). A total of 338,318 sentinel chicken blood samples were tested and 7,340 (2.2%) WNV seroconversions were reported from 38 counties (Fig 4 and S7 Table). Beginning in 2008, the number of sentinel flocks decreased annually, and by 2018, the number of samples tested (9,871) had decreased by 70% since 2003 (33,830) (Fig 4). Excluding 2003, the annual percentage of flocks with at least one chicken undergoing WNV seroconversion ranged from 28% (2010) to 67% (2005).

From 2004 through 2018, chickens annually seroconverted in all three regions. On average, the first seroconversion occurred on June 15 in southern California, whereas in the Central Valley and other California, on average the first seroconversions occurred on July 2 and July 31, respectively. Ninety-five percent of all seroconversions occurred from July through October, with the most in August (1,240, 16.9%). Only twenty seroconversions (0.3%) occurred between December 1 and March 31, but almost all samples (328,400, 97.1%) were collected from April through November. Most seroconversions (3,647, 49.7%) occurred in the Central Valley, even though these counties maintained fewer total flocks, and fewer flocks per county than counties in southern California. The flock seroconversion rate varied by region and year, although in every year except 2004, 2008, and 2017, Central Valley counties reported the highest percentage of flocks that had >1 seroconversions (Fig 5). WNV seroconversions were not detected in three counties that maintained sentinel flocks for at least five of the sixteen years (consecutively or non-consecutively): Monterey, San Mateo, and Santa Cruz counties.

### Non-equine mammal surveillance

WNV prevalence from 2004 through 2018 in tree squirrels ranged from 10% (2009; 10 positive among 103 tested) to 57% (50/88) in 2004. The most common species reported dead and tested were the non-native fox squirrel *(Sciurus niger)* (647; 31% prevalence), non-native eastern gray squirrel *(Sciurus carolinensis)* (300; 12%), and native western gray squirrel *(Sciurus griseus)* (315; 18%). Most squirrels were not identified to species unless they were collected for WNV testing; therefore, counts were reported only among those tested [61].

From 2004 through 2013, limited WNV testing was done among other animals with suspected WNV infection. This resulted in WNV detections in alpacas (*Vicugna pacos*) (2/3 positive), llamas (*Lama glama*) (1/1), jackrabbits (Genus *Lepus*) (1/3), other rabbits (Family Leporidae) (2/18) and sheep (*Ovis aries*) (3/3).

## Discussion

Before and since the arrival of WNV, California has maintained a comprehensive arbovirus surveillance program that pairs enzootic surveillance (mosquitoes, dead birds, and sentinel chickens) with human surveillance to characterize and minimize arboviral risk in humans. In most years, the risk of WNV disease was greatest in California’s Central Valley compared to southern California and elsewhere in the state (other California); the Central Valley usually reported the highest WNV disease incidence, mosquito MIR, and chicken flock seroconversion rate. The percentage of WNV-positive dead wild birds in the Central Valley was more variable, likely because public reporting of these birds was lower and more variable in some rural agricultural areas with the highest WNV disease incidence. This elevated WNV activity in the Central Valley reflected the historically high risk of arboviral disease in the region [65,66]. However, Los Angeles County reported more human cases and enzootic activity than anywhere else in California (and most other states), likely due to the county’s large human population (>10 million people), increased *Cx*. *quinquefasciatus* populations compared with elsewhere in the state, and abundant competent avian host populations (American crows, house finches, and house sparrows)[67]. WNV-positive mosquitoes were also usually detected both earlier and later in the year in southern California relative to the Central Valley, reflecting a longer WNV transmission season in warmer Southern California.

Most WNV-positive mosquitoes were collected between June and October (98%), with peak mosquito activity occurring in August. The surveillance program provided data demonstrating that WNV remained active in California throughout the year, with dead birds and positive mosquitoes detected every month. Of note, *Culex spp*. mosquitoes do not always enter reproductive diapause in the winter in southern California, potentially enabling year-round transmission [31,68]. Similarly, almost 95% of human disease occurred between June and October and peaked in August. However, a small number of WNV disease cases developed symptoms in winter in areas that did not report other WNV detections (i.e., the detections of overwintering WNV in birds did not correspond with the locations of these human infections). Due to the non-specific nature of arboviral diagnostic assays and the persistence of IgM, we suspect that these individuals’ symptoms were not due to acute WNV disease, and that their clinical manifestation represented another disease’s etiology, an anamnestic immune response, or a false-positive assay result [69]. Many human cases reported in the winter had serological assay results that were very close to diagnostic cutoff values [S. Messenger, personal communication], which could be suggestive of false positive / non-specific immune responses, or the coincidental detection of WNV IgM in cases with other underlying etiologies. Further, several immunocompetent WNV case-patients who have undergone sequential, annual testing for WNV have shown IgM elevated above the clinical cutoff for several years after symptom onset [S. Messenger, personal communication].

Statewide, the proportion of non-neuroinvasive cases reported each year has decreased from a high of 63% of reported cases (2006) to a low of 22% (2016). In California, this is likely due to reductions in testing among cases with mild to moderate symptoms, or diminished interest among both patients and providers in obtaining definitive diagnoses of febrile illness. There is no evidence of any changes in the virulence or pathogenicity of the virus itself that could contribute to more neuroinvasive infections. These changes in reporting underscore the importance of using WNND to estimate the incidence of WNV disease. There are an estimated 30–70 non-neuroinvasive disease infections for every neuroinvasive case [70–72], extrapolating to 122,000–285,000 non-neuroinvasive WNV disease infections in California from 2003 through 2018 and a cumulative incidence of approximately 327–765 WNV infections / 100,000 residents. Averaging across 16 years, this annual incidence between 19 and 45 West Nile non-neuroinvasive disease infections / 100,000 residents, approaches the reported incidence of Lyme disease in some high-incidence northeastern states [73]. Given that only an estimated 20% of WNV infections become symptomatic, these infections represent a mere fraction of all WNV infections that have occurred; the true number of WNV infections is likely much higher.

While striving for consistency in sampling, testing, and surveillance since the virus was first detected in California, several fundamental changes have been made to human WNV surveillance. Outside of modifications made to the CSTE case definition, which may have influenced WNV human disease reporting, the development and integration of CalREDIE and other electronic tools for infectious disease surveillance have dramatically improved data collection, timeliness and quality, further enhancing our understanding of WNV disease. Unfortunately, it is not possible to control for changes in the case definition of WNV disease; however, our ability to predict and estimate the risk of WNV transmission to humans will continue to improve as more years of data accumulate in these databases. Some limitations and biases in passive infectious disease surveillance have already been extensively discussed elsewhere [74–82]. Unlike active WNV blood donor surveillance, in which all blood donations are screened for WNV, passive surveillance of patients with symptomatic WNV depends on care availability and provider recognition, yielding a difficult to define epidemiologic sampling frame. Further, while standardized electronic case forms are currently used, data collection has varied among jurisdictions as public health resources have fluctuated and may depend on the availability of patients or their families to provide information to case investigators. There are also key health equity concerns that could influence our understanding of the epidemiology of WNV. Little is known about arboviral disease incidence in people with insecure housing or among migrant farmworkers. Both of these medically underserved populations are at increased risk for mosquito bites due to the amount of time they spend outside, particularly at dusk and dawn [83–86]. Additional equity issues related to healthcare-seeking behavior, particularly in rural and medically underserved populations, and the availability of health insurance, also likely influence our understanding of the epidemiology of WNV disease.

In response to new research and other evolving best practices, enzootic surveillance methods, sampling intensity, and laboratory testing has also improved since 2003. For example, the utility of horses as sentinels for WNV activity has changed since the virus’ introduction. The decrease in the number of reported horse infections is directly related to widespread annual vaccination (for which robust data are not available). However, the practice is widespread. In addition, pastured horses are also likely to have been naturally infected, developing protective immunity independent of vaccination [87].

Importantly, resource availability directly influences surveillance activities conducted by VCAs. For instance, mosquito, chicken, and wild bird testing was initially centralized, with all samples being tested by DART or VRDL, but gradually, several VCAs have begun testing specimens in their own laboratories. Rapid assay kits also were used in some years by VCAs to test birds and mosquitoes but due to decreased sensitivity were phased out [53]. In contrast, molecular testing of avian oral swabs on nucleic acid preservation cards was found to be highly sensitive and specific, [48,54,88] and allowed for avian testing without the need for necropsy. Importantly, to ensure consistency among testing laboratories, the previously described proficiency panels were developed and annually provided to VCAs by DART.

Although mosquito pool testing has increased substantially since 2003, sampling effort and spatial coverage has varied widely across the state; thus, the intensity of surveillance conducted by VCAs should be considered when comparing MIRs among counties and regions. VCAs continue to use the MIR within their jurisdictions to help determine WNV transmission risk and guide mosquito control activities (12). In areas with robust mosquito sampling, increased MIRs tended to correlate with increases in reported human disease shortly thereafter, especially when restricting MIR estimates to the peak WNV season (July to October).

Statewide, the MIR was highest in urban *Cx*. *pipiens* complex mosquitoes. In California, this complex consists of *Cx*. *quinquefaciatus* in southern California, and *Cx*. *quinquefasciatus/Cx*. *pipiens* intergrades in the Central Valley [30]. These mosquitoes are collected most effectively by gravid female traps that sample mosquitoes which have previously blood fed; therefore, this part of the population is more likely to be infected with WNV than those collected host-seeking via CO_2_ baited traps. The MIR for *Cx*. *tarsalis* in the Central Valley was more than three times higher than the MIR in southern California and other California. Importantly, MIRs should be considered along with the relative abundance of each species, and in recent years many VCAs have begun to use the vector index in conjunction with MIR for mosquito control decisions [88]. The vector index is an estimate of the abundance of infected mosquitoes per trap-night. Interestingly, ornithophagic and laboratory-vector competent *Cx*. *stigmatosoma* and *Cx*. *thriambus* had MIRs comparable to the more recognized *Culex* vector species, but *Cx*. *stigmatosoma* and *Cx*. *thriambus* were relatively patchier in their distributions, or less abundant and more difficult to sample, respectively. Only 47 (0.15%) WNV positive pools were detected in non-*Culex* species, although most testing has focused on *Culex spp*. after initial testing showed that species which fed predominantly on mammals were infrequently infected with WNV. Invasive potential bridge vector species such as *Aedes aegypti* and *Ae*. *albopictus* that have been spreading in California since 2013 and 2011, respectively, were rarely positive. During the time period encompassed by this report only a single (1/361; 0.3%) *Ae*. *albopictus* and seven (7/2564; 0.3%) *Ae*. *aegypti* pools tested positive for WNV.

In contrast to mosquito testing, the number of sentinel chicken flocks has decreased substantially statewide since 2003. From 2004 through 2018, the number of flocks located in each region decreased by 66% (southern California), 59% (Central Valley), and 18% (other California). Many VCAs have diverted resources from sentinel chicken sampling to expanded mosquito sampling and testing to enhance early WNV detection. Dead bird reports, which rely upon public awareness of their role in virus transmission and their willingness to report them, have also declined statewide recently, perhaps due to local declines in WNV activity (both true and perceived), diminished outreach campaigns, or public fatigue in reporting dead wild birds. In addition, since 2003 some VCAs have refined their sampling criteria to limit collection and testing to bird species which are the best WNV indicators (corvids, raptors, and songbirds) [53,89–91]. VCA participation in the program has declined slightly, however most counties in the Central Valley and Southern California continue to participate.

Despite these limitations, reliance on human disease surveillance alone is inadequate to direct adult mosquito control and limit WNV transmission risk given delays from spillover transmission to human infection, symptom onset, diagnostic testing, case investigation, and reporting to public health authorities. Enzootic surveillance indicators have consistently predated human disease in California, while rapid detection and monitoring of WNV activity in enzootic indicators has prompted mosquito abatement response activities to reduce and (ideally) interrupt enzootic amplification and thereby mitigate human risk. Enhanced data management and sharing practices implemented in California have further improved data quality and encouraged collaboration while reducing operating costs.

California’s comprehensive WNV surveillance system and diverse ecological landscape present a wealth of opportunities for studies of viral phylogenetics. Since WNV arrived in the state, it has continued to evolve [22,92]. The WN02 strain has been the predominant strain in North America since 2003 [19,92,93], whereas the SW03 strain arose from WN02 and differs by two non-structural mutations [21,94]. The mutations that characterize these genotypes appear to be favored over the initial NY99 strain due to their continued co-circulation and relative stability over time [22,92]. Overall, WN02 has been the more prevalent strain in California, and the relative prevalence of WN02 versus SW03 differs regionally, with more frequent detections of SW03 in southern California compared to northern California [22,92]. WNV has been introduced into California on at least five distinct occasions [22], and many mutations have arisen during that time. Specific WNV variants have been detected repeatedly across multiple years and there is minimal mixing of California WNV genomes with those from the eastern U.S. [92], which suggests that local transmission and overwintering within California, rather than long-range dispersal events, are the primary mechanism for viral persistence between years.

California’s vector-borne disease surveillance program serves as the basis for integrated vector management throughout the state. Surveillance data are used by local vector control agencies to guide mosquito prevention and control activities, and elevated disease risk prompts an escalating sequence of intensifying intervention activities as described in the California Mosquito-borne Surveillance and Response Plan [14]. Local agencies regularly evaluate risk of WNV transmission using CalSurv charts, maps, and other tools, and communicate these findings to their local constituents. Collectively, these activities can help to minimize WNV morbidity and mortality in California residents and visitors.

As mosquitoes and mosquito-borne viruses continue to expand across the globe and impact California, it is imperative that local and state entities remain vigilant to the threat of arboviral disease and work together with neighboring states and countries to improve our understanding of disease ecology and epidemiology.

## Conclusion

California has consistently more WNV activity than any other state in the continental United States. The state’s multi-faceted arbovirus surveillance program has yielded a uniquely comprehensive dataset that offers a wealth of information to improve our understanding of arboviral dynamics. Geographic and temporal patterns of WNV activity are influenced by climate, the distribution and abundance of mosquito vectors and reservoir birds, as well as mosquito control and public health efforts. Surveillance using mosquitoes, dead birds, and sentinel chickens is employed throughout the state to guide WNV disease prevention efforts.

WNV is endemic throughout California, but its distribution and seasonal activity patterns vary regionally within the state. The majority of WNV enzootic activity and the highest WNV disease incidence was in the state’s Central Valley, followed closely by southern California. Less than five percent of all WNV activity occurred outside of these regions (other California). Annual changes in the geographic distribution of WNV outbreaks complicates intervention and public messaging regarding disease risk. Despite this, WNV has been detected in every California county, with human disease having been reported among residents of most. It remains critical that all Californians remain informed and vigilant to protect themselves from mosquito bites and therefore WNV infection.

Despite robust surveillance and control, it remains critical that mosquito bite prevention via repellents and protective clothing continue to be emphasized. In addition, local and state public health partners must continue statewide human surveillance of WNV and other emergent arboviruses, while investing in and conducting robust, cost-effective enzootic surveillance to guide mosquito-borne disease prevention and control activities.

## Supporting information

S1 Table Symptomatic (S) and asymptomatic (A) West Nile virus (WNV) infections, and 16-year cumulative incidence of WNV disease by county of residence, California, 2003–2018.
(XLSX)

S2 Table West Nile virus mosquito testing by county, California, 2003–2018. (XLSX)

S3 Table West Nile virus minimum infection rate among *Culex spp*. mosquitoes by county, California, 2003–2018. (XLSX)

S4 Table Mosquito species tested for West Nile virus in California, 2003–2018. (XLSX)

S5 Table Birds collected via the California Dead Bird Species Program by county and year, 2003–2018. (XLSX)

S6 Table Bird species with at least 99 individuals testing positive and WNV testing results among birds collected in California, 2003–2018. (XLSX)

S7 Table Number of sentinel chicken flocks and WNV seroconversions by county and year, California, 2003–2018. (XLSX)
